# The Relationship between AGAMOUS and Cytokinin Signaling in the Establishment of Carpeloid Features

**DOI:** 10.3390/plants10050827

**Published:** 2021-04-21

**Authors:** Andrea Gómez-Felipe, Daniel Kierzkowski, Stefan de Folter

**Affiliations:** 1 Unidad de Genómica Avanzada (UGA-Langebio), Centro de Investigación y de Estudios Avanzados del Instituto Politécnico Nacional (CINVESTAV-IPN), Irapuato CP 36824, Guanajuato, Mexico; andrea.gomez.felipe@umontreal.ca; 2 Department of Biological Sciences, Plant Biology Research Institute, University of Montreal, Montreal, QC H1X 2B2, Canada; daniel.kierzkowski@umontreal.ca

**Keywords:** AGAMOUS, cytokinin signaling, carpel, gynoecium, transcription factors, CRC, SPT, SHP2, type-B ARR

## Abstract

Gynoecium development is dependent on gene regulation and hormonal pathway interactions. The phytohormones auxin and cytokinin are involved in many developmental programs, where cytokinin is normally important for cell division and meristem activity, while auxin induces cell differentiation and organ initiation in the shoot. The MADS-box transcription factor AGAMOUS (AG) is important for the development of the reproductive structures of the flower. Here, we focus on the relationship between AG and cytokinin in *Arabidopsis thaliana*, and use the weak *ag-12* and the strong *ag-1* allele. We found that cytokinin induces carpeloid features in an AG-dependent manner and the expression of the transcription factors *CRC*, *SHP2*, and *SPT* that are involved in carpel development. AG is important for gynoecium development, and contributes to regulating, or else directly regulates *CRC*, *SHP2*, and *SPT*. All four genes respond to either reduced or induced cytokinin signaling and have the potential to be regulated by cytokinin via the type-B ARR proteins. We generated a model of a gene regulatory network, where cytokinin signaling is mainly upstream and in parallel with AG activity.

## 1. Introduction

Angiosperms produce flowers during their reproductive phase. Floral organs develop from pluripotent stem cells located in specialized tissues called floral meristems (FMs) [1]. In Arabidopsis, each FM gives rise to four types of organs arranged in concentric whorls: four sepals in whorl 1, four petals in whorl 2, six stamens in whorl 3, and one gynoecium that consists of two fused carpels in whorl 4 [2,3]. Many genetic studies over the last 30 years have identified important transcription factors that orchestrate floral development (reviewed in: [4,5,6,7,8]. One very well-known transcription factor is AGAMOUS (AG) of the MADS-box family, which fulfills the C-function of the ABC model specifying the reproductive organs [9,10,11]. AG controls several processes such as stamen identity, carpel identity, FM determinacy, microsporogenesis, organ maturation, and prevents the misexpression of the A-function genes in the third and fourth whorls [12]. The lack of AG activity causes alterations in the third and fourth whorl organs: petals develop at the positions of stamens, and at the position of the gynoecium, the FM remains active and a new flower develops. This process repeats itself indeterminately, resulting in the flower-in-flower phenotype [9,10,13]. Constitutive expression of AG results in carpeloid sepals and staminoid petals in the first and second whorl [14]. It is known that AG activity inhibits AP2 function and, vice versa, AP2 function represses AG activity [15]. For instance, whorls 1 and 2 of *ap2* mutant flowers have carpeloid features due to ectopic AG activity [9,12,13]. These data support that carpeloid features are regulated through AG targets. However, flowers of the *ap2-2 ag-1* double mutant have still carpeloid features. Based on genetics, it has been shown that *CRABS CLAW* (*CRC*) and *SPATULA* (*SPT*) also promote carpel tissue development in parallel with *AG* [16,17,18]. In the *ag-1* null mutant no carpeloid features are observed due to ectopic AP2 function, and AP2 represses CRC and SPT function [16]. Furthermore, the *AG* paralog *SHATTERPROOF2* (*SHP2*) gene is also able to induce carpel development, in an AG-independent manner [19,20]. In the quadruple mutant *ap2 ag shp1 shp2*, all carpeloid features were absent. Furthermore, ectopic *SHP2* expression can complement the *ag-1* mutant and causes ectopic carpeloid structures, something that CRC and SPT do not [19,21,22]. Based on genetics, it can be suggested that AG and SHP1/2 redundantly regulate *SPT* and *CRC* expression [16,19,23]. The transcription factors *CRC* and *SHP2* are direct targets of AG [24,25]. *SPT* has not been demonstrated to be a direct target of AG. Altogether, these data indicate that AG is important, though not fully required to generate all carpel properties. 

Phytohormones such as auxin and cytokinin are important factors during plant development. This holds also for carpel initiation and gynoecium development (for reviews: [8,26,27,28,29,30,31,32,33]). In general, cytokinin is important for meristem formation and maintenance, and auxin is important for organ differentiation [34,35]. It is well known that phytohormones interact to maintain their fine-turned regulatory functions, and auxin and cytokinin are also referred to as the ´yin and yang´ of plant development. In recent years, substantial progress has been made in elucidating the molecular mechanisms of auxin and cytokinin signaling during carpel initiation and early gynoecium development (e.g., [8,26,36,37,38,39,40,41,42,43,44,45,46,47,48,49,50,51,52,53]).

However, there is still a gap in understanding how hormones and transcription factors interact. A few recent studies related AG and cytokinin signaling [42,43,44]. It has been reported that AG activity has a negative effect on cytokinin signaling [42,44]. In another study, it has been shown that the cytokinin signaling type-B ARR proteins physically bind to the second intron of AG to control carpel regeneration from calli [43]. Here, we further addressed the relationship between AG and cytokinin signaling. Furthermore, we also addressed the involvement of the three known carpel development promoting transcription factors, CRC, SHP2, and SPT. We found that cytokinin induces carpeloid structures in an AG-dependent manner and the expression of the transcription factors *CRC*, *SHP2*, and *SPT*, which are involved in carpel development. Furthermore, *AG*, *CRC*, *SHP2*, and *SPT* respond to altered cytokinin signaling and have the potential to be directly regulated by type-B ARR proteins. The cytokinin-mediated induction of *AG* and *SPT* seems to be importantly, or entirely, dependent on ARR1, ARR10 and/or ARR12. We propose a model of a gene regulatory network where cytokinin signaling acts mainly upstream and in parallel with AG activity during early stages of flower development.

## 2. Materials and Methods

### 2.1. Plant Growth Conditions

*Arabidopsis thaliana* plants used in this study were the *ag-12* weak mutant (T-DNA insertion line SALK_014999) in the Col-0 background [54], the *ag-1* null mutant in the L*er* background [10], the type-B ARR *arr1-3 arr10-5 arr12-1* (*arr1 arr10 arr12*) triple mutant (CS39992; [55]), and the wild type accessions Col-0 and L*er*. Seeds were germinated on soil under long-day conditions (16/8 h, light/dark) in a growth chamber at 22°C. One week after germination, plants were transferred to the greenhouse with a temperature range from 22 to 28 °C, with long-day conditions (13/11 h, light/dark) and natural light.

### 2.2. Hormone Treatments

One week after bolting, the *ag-12* mutant, the *ag-1* mutant and wild type inflorescences were treated for ten consecutive days with BAP solution (100 µM 6-benzylaminopurine (Sigma, Toluca, Mexico) and 0.015% (*v/v*) Silwet L-77) (Lehle Seeds, Round Rock, US) or mock solution (0.015% (*v/v*) Silwet L-77 in water) by submerging the inflorescence for ~1 min.

### 2.3. Scanning Electron Microscopy

One week after the last BAP or mock treatment, flowers from BAP and mock were scanned using a Zeiss EVO40 environmental scanning electron microscope (Carl Zeiss; Oberkochen, Germany) with 25 kV beam, and the signal was collected using the SE detector. Each plant tissue was collected and directly observed in the microscope.

### 2.4. qRT-PCR Analysis

For qRT-PCR analysis, *ag-12*, *ag-1* and wild type inflorescences were treated once with BAP or mock solution by submerging the inflorescences for ~1 min. 24 h later, young floral buds from 10 individual plants for each line were harvested and frozen in liquid nitrogen. Three biological replicates were sampled. Immediately after harvesting, total RNA was extracted using the Quick-RNA MicroPrep Kit (Zymo Research, Irvine, CA, USA). Samples were treated with DNase I, included in the kit. Reverse transcription and amplification were performed using a KAPA SYBR FAST One-Step qRT-PCR Kit (kapa Biosystems, Cape Town, South Africa). qRT-PCR was performed on a StepOneTM thermocycler (Applied Biosystems, Foster City, CA, USA). Target gene expression levels were normalized to ACTIN 2 and calibrated to the average ΔCt of the wild type. Data was analyzed using the 2^−ΔΔC^_T_ method [56]. Primers used are listed in the supplementary Table S1.

## 3. Results and Discussion

### 3.1. Exogenous Cytokinin Induces Carpeloid Features in an AG-Dependent Manner

**Figure 1:**
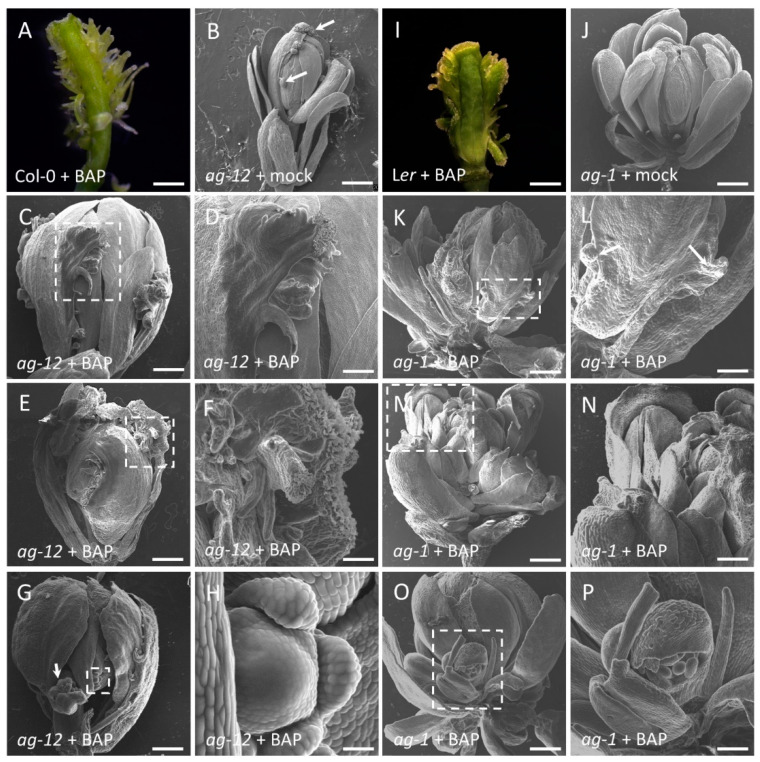
Cytokinin-induced carpeloid structures and secondary floral buds in flowers of plants lacking AG activity. Scanning electron micrographs of floral phenotypes of *ag* mutant plants treated with mock or cytokinin (100 µM BAP) solution; pictures are of one week after the treatment. (**A**) Micrograph of a gynoecium Col-0 treated with cytokinin solution. (**B**) Mock-treated *ag-12* flower showing ectopic ovules and stigmatic papillae structures (as indicated by the white arrows). (**C**–**H**) BAP-treated *ag-12* flowers, stigmatic and carpeloid structures are visible. (**D**,**F**) Close up from dotted white square in (**C**) and (**E**), respectively. (**G**) BAP-treated *ag-12* flower showing floral buds (as indicated by the white arrow). (**H**) Close up from dotted white square in (**G**) showing a floral meristem. (**I**) Micrograph of a gynoecium L*er* treated with cytokinin solution. (**J**) Mock treated *ag-1* flowers without carpeloid structures (some sepals and petals were removed). **(K**–**N)** BAP-treated *ag-1* flowers (some sepals and petals were removed) showing only some proliferating tissue (as indicated by the white arrows). (**L**,**N**) Close up from dotted white square in (**K**) and (**M**), respectively. (**O**) BAP-treated *ag-1* flower showing a floral bud (as indicated by the dotted white square). (**P**) Close up from dotted white square in (**O**) showing a floral bud. (**D**,**F**,**H**,**L**,**N**,**P**) Close up from dotted white square with a higher magnification. Scale bars: (**B**–**G**, **J**–**O**) 200 µm, (**A**,**I**,**P**) 100 µm, (**H**) 30 µm.

We have previously shown that cytokinin is involved in gynoecium development [8,39,45,49,53]. Repeated exogenous applications of cytokinin (6-benzylamonopurine; BAP) to wild type inflorescences induces ectopic proliferative tissue from the replum, which has at its tip stigmatic-like characteristics (Figure 1A,I; [49]). We decided to analyze the relationship between the transcription factor AGAMOUS (AG) and cytokinin signaling in the establishment of carpeloid features using the same pharmacological assay.

To achieve this, we used two recessive mutants, *ag-12* and *ag-1*, that show replacement of stamens and carpels by petals and sepals, resulting in a flower within a flower phenotype, characteristic of AG loss-of-function phenotypes [9,10,42]. *ag-12* and *ag-1* are different in phenotypic severity, with *ag-12* being a weaker allele compared to the strong *ag-1* null allele. In the absence of exogenous BAP, *ag-1* does not present any carpeloid structures (Figure 1J), while *ag-12* presents some ectopic ovules and stigmatic papillae (Figure 1B), likely due to the fact that this mutant still produces full length transcripts, though at reduced levels compared to wild-type plants [57]. Furthermore, *ag-12* shows an elongated pedicel resulting in a separation of the internal flowers, which has been attributed to *ag-12* and *ag-1* being in different genetic backgrounds [10].

Upon exogenous BAP treatment, we observed in the *ag-12* mutant proliferation displaying stigmatic papillae at the apex (85% of 80 observed flowers, Figure 1C–F). In BAP-treated *ag-1* plants, 75% of 80 observed flowers showed marginal outgrowth in the sepals, but no stigmatic papillae or any other carpeloid structure (Figure 1K–N). In both mutant alleles, we observed the formation of secondary floral buds in the axil of the sepals (Figure 1G,H,O,P), which has been reported for the strong *ag-1* allele [46]. As expected, the mock treatment did not induce any carpeloid structures in *ag-12* and *ag-1*, nor induced the formation of secondary floral buds (Figure 1B,J). These results suggest that the residual AG activity in *ag-12* is sufficient to induce carpeloid structures, and that BAP-treatment induces tissue proliferation, as it does in the wild type (Figure 1A,I), and this tissue acquires carpeloid characteristics only in the presence of AG.

### 3.2. Cytokinin Induces AG Targets Involved in Gynoecium Development

**Figure 2:**
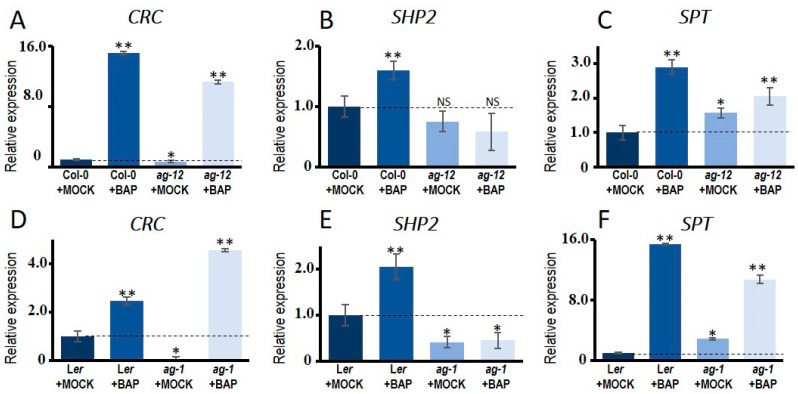
Expression levels of *CRC, SPT* and *SHP2* in *ag* mutant backgrounds and wild type. (**A**–**F**) qRT-PCR expression data for *CRC*, *SPT* and *SHP2* in floral buds after 24 h of cytokinin-treatment (100 µM BAP) or mock-treatment in the *ag-12* mutant (**A**–**C**) and in the *ag-1* mutant (**D**–**F**). The results were obtained for three independent biological replicates with three technical replicates for each one. One-way ANOVA was used to evaluate the significant differences. Significant values are indicated as follow: (*) *p* ˂ 0.05, (**) *p* ˂ 0.01, not significant (NS), compared to mock-treated wild type.

Three genes have been reported to induce carpel features independently of AG: CRABS CLAW (CRC), SPATULA (SPT), and SHATTERPROOF 2 (SHP2) [16,19]. We wondered whether one or more of these genes could be induced by cytokinin, thus explaining the observed carpeloid features in the *ag-12* BAP-treated flowers. In wild type flowers, either Col-0 or L*er*, all three genes are induced 24 h after BAP treatment, compared to their corresponding mock controls (Figure 2A–F). In the mock-treated *ag-1* null mutant flowers, *CRC* and *SHP2* showed significantly reduced expression compared to the mock-treated wild type flowers (Figure 2D,E). In the weak *ag-12* mutant, *CRC* and *SHP2* showed a slightly reduced expression, though *SHP2* reduction is not significantly different, compared to the mock-treated wild type flowers. This suggests that AG induces the expression of *CRC* and *SHP2*, and the less reduced expression in *ag-12* is probably due to the residual AG activity in this mutant. On the other hand, the expression of *SPT* was increased in both *ag* mutants compared to the wild type, suggesting that *SPT* expression is independent of AG (Figure 2C,F). In the BAP-treated *ag* mutants, *CRC* and *SPT* were induced compared to the wild type, while *SHP2* expression was not significantly altered by BAP.

This suggests that *SHP2* response to cytokinin is dependent on the presence of AG, and that *CRC* and *SPT* can respond to cytokinin even with a reduced or absent AG function. Furthermore, these results suggest that the carpeloid structures observed in the BAP-treated weak *ag-12* mutant could be due to mainly the presence of residual AG activity, and perhaps in addition to the presence of *SHP2* expression. The BAP-induction of *CRC* and *SPT* expression alone is not sufficient, because in the strong *ag-1* allele no BAP-induced carpeloid features were observed.

### 3.3. Different Effects of Type-B ARR Proteins on Carpel Promoting Genes

**Figure 3:**
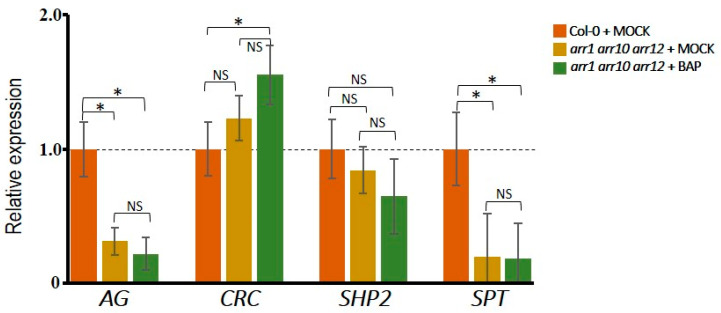
Expression levels of *AG*, *CRC*, *SPT* and *SHP2* in the type-B *arr1 arr10 arr12* triple mutant. qRT-PCR expression data for *AG*, *CRC*, *SPT* and *SHP2* in floral buds after 24 h of cytokinin-treatment (100 µM BAP) or mock-treatment in the type-B *arr1 arr10 arr12* triple mutant. The results were obtained for three independent biological replicates with three technical replicates for each one. One-way ANOVA was used to evaluate the significant differences. Significant values are indicated as follow: (*) *p* ˂ 0.05, not significant (NS), compared to mock-treated wild type.

The cytokinin signaling pathway affects gene expression via the type-B ARR transcription factors. We hypothesized that the observed positive effect of cytokinin on *CRC*, *SPT* and *SHP2* expression could be regulated through the type-B cytokinin response regulators (type-B ARRs). Therefore, we tested by qRT-PCR the expression of *CRC*, *SPT* and *SHP2* in the *arr1 arr10 arr12* triple mutant, which is severely affected in cytokinin signaling [58,59,60,61]. As a positive control, we evaluated the expression of *AG*, which has already been tested previously in the *arr1 arr10 arr12* triple mutant [43]. As reported by Rong and colleagues, *AG* expression was reduced in the *arr* triple mutant (Figure 3). Furthermore, also *SPT* showed a strong reduction in expression (Figure 3). In contrast, the AG direct targets *CRC* and *SHP2* showed no significant change in expression, meaning they are also regulated by other genes (Figure 3). Next, we analyzed the gene expression after 24 h of BAP-treatment. Interestingly, though to be expected, none of the four genes responded to cytokinin in the *arr1 arr10 arr12* triple mutant when compared to the mock-treatment of the *arr* triple mutant (Figure 3). Note, a slight but statistically significant BAP-induction of *CRC* expression was observed in the *arr* triple mutant when compared to mock-treated wild type plants. This could suggest that other type-B ARR proteins might regulate *CRC* expression as well. 

As described above, expression of *CRC*, *SPT*, and *SHP2* in wild type plants is induced upon cytokinin-treatment (Figure 2). So, this may be due to direct or indirect regulation by the transcription factors ARR1, ARR10, and ARR12. Recently, it has been shown that *AG* is a direct target of ARR1 and ARR10 [43]. Additionally, based on analysis of ChIP-seq data of ARR1, ARR10 and ARR12 [61], we observed type-B ARR binding to the *CRC*, *SPT* and *SHP2* promoter regions (Figure S1). It is noteworthy that seedlings were used to generate the ChIP-seq data [62], however, clear signals can be observed in the regulatory regions of *AG*, *CRC*, *SPT*, and *SHP2* (Figure S1). The ARR1 binding to the second intron of *AG* that has been previously reported using ChIP by Rong and colleagues [43], can clearly be observed in the ChIP-seq tracks (Figure S1).

Altogether, this indicates that *AG*, *CRC*, *SPT*, and *SHP2* have the potential to be directly regulated by type-B ARR proteins. The expression levels of *AG* and *SPT* seem to be more dependent on type-B ARRs ARR1, ARR10 and ARR12 function than *CRC* and *SHP2*. Concerning the ability to respond to exogenous cytokinin, all four genes are dependent on the type-B ARRs ARR1, ARR10 and ARR12 proteins. Finally, the *SHP2* gene responds to cytokinin in an AG-dependent manner and has clear ChIP-seq signals for the type-B ARR proteins, suggesting that *SHP2* might be cooperatively regulated by AG and ARRs in this condition.

### 3.4. Model of Regulatory Network between AG and Cytokinin

**Figure 4:**
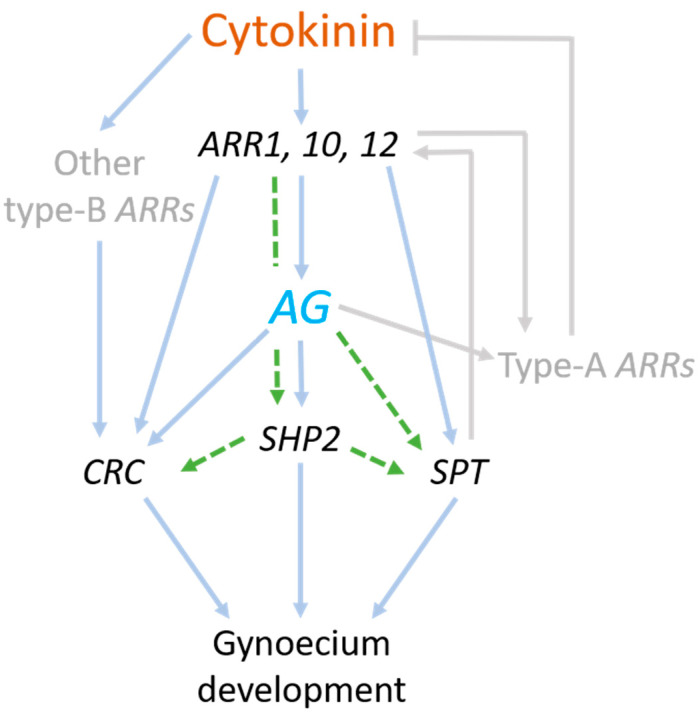
Model of a gene regulatory network of the relationship between *AG*, *CRC*, *SPT*, *SHP2*, and cytokinin signaling during early gynoecium development. AG is important for gynoecium development, and contributes to regulating or directly regulates *CRC*, *SHP2*, and *SPT*. All four genes respond to either reduced or induced cytokinin signaling. Cytokinin-mediated induction of *AG* and *SPT* is dependent on type-B ARR proteins (ARR1, ARR10, ARR12). The *SHP2* gene responds to cytokinin in an AG-dependent manner and might be cooperatively regulated by AG and ARR1 in this condition. AG regulates type-A ARR genes, which negatively feedbacks to cytokinin signaling (in grey colored lines); a negative feedback loop is important for homeostasis. The second negative feedback loop is activated by the type-B ARR proteins. Green dashed lines are suggested interactions and might be direct or indirect regulatory interactions. Not indicated, but all transcription factors are also regulated by other factors.

We generated a model of a possible gene regulatory network where cytokinin signaling acts mainly upstream and in parallel with AG activity during carpel differentiation (Figure 4). AG is important for gynoecium development, and contributes to regulating or directly regulates *CRC*, *SHP2*, and *SPT*. All four genes respond to either reduced or induced cytokinin signaling. Cytokinin-mediated induction of *AG* and *SPT* is dependent on the type-B ARR1, ARR10, and ARR12 proteins. Furthermore, cytokinin-induced expression of *CRC*, *SHP2*, and *SPT* is dependent on type-B ARR protein activity, and *SHP2* cytokinin-induced expression is also dependent on AG activity. Results also suggest that *CRC* is probably also regulated by other type-B ARR proteins. In addition, it is known that AG regulates type-A ARR genes, which negatively affect cytokinin signaling, meaning that cytokinin signaling is also downstream of AG [42,62]. It is clear that various pathways in parallel regulate carpel differentiation by controlling the hormonal balance between cytokinin and auxin. In the moment when cytokinin signaling goes down in the FM, probably the auxin pathway takes over to induce carpel differentiation, which can happen by the activity of AG and CRC [41,62], including SPT [39]; all induce auxin biosynthetic genes. In addition, the direct AG target *ETT*, which also responds to auxin, represses cytokinin signaling [44]. On the other hand, during carpel differentiation, SPT activates the type-B ARR proteins for cytokinin signaling [39]. Likely, all these regulatory interactions are dynamic, and depend on the developmental time, tissue type, and transcription factor complexes. Future research will elucidate more details on how AG and other transcription factors are involved in cytokinin and auxin homeostasis to ensure proper carpel initiation and differentiation.

## 4. Conclusions

We found that cytokinin induces carpeloid features in an AG-dependent manner and the expression of the transcription factors *CRC*, *SHP2*, and *SPT* that are involved in carpel development. AG is important for gynoecium development, and contributes to regulating, or else directly regulates *CRC*, *SHP2*, and *SPT*. All four genes respond to either reduced or induced cytokinin signaling and have the potential to be regulated by cytokinin via the type-B ARR proteins. We generated a model of a gene regulatory network, where cytokinin signaling is mainly upstream and in parallel with AG activity.

## Data Availability

No new data were created or analyzed in this study. Data sharing is not applicable to this article.

## Supplementary Materials

The following are available online at https://www.mdpi.com/article/10.3390/plants10050827/s1, Figure S1. Type-B ARR binding events in the *AG*, *CRC*, *SPT* and *SHP2* regulatory regions. Table S1. Oligonucleotides used in this study.
