# Supplementary material for: The Relationship between AGAMOUS and Cytokinin Signaling in the Establishment of Carpeloid Features
Source: Plants (Basel). 2021 Apr 21;10(5):827. doi: 10.3390/plants10050827 (PMC8143136; doi:10.3390/plants10050827)
Supplement: Supplementary file 1 [file plants-10-00827-s001.zip › plants-1138997-supplementary.pdf]

plants

MDPI

**Supplementary Materials**

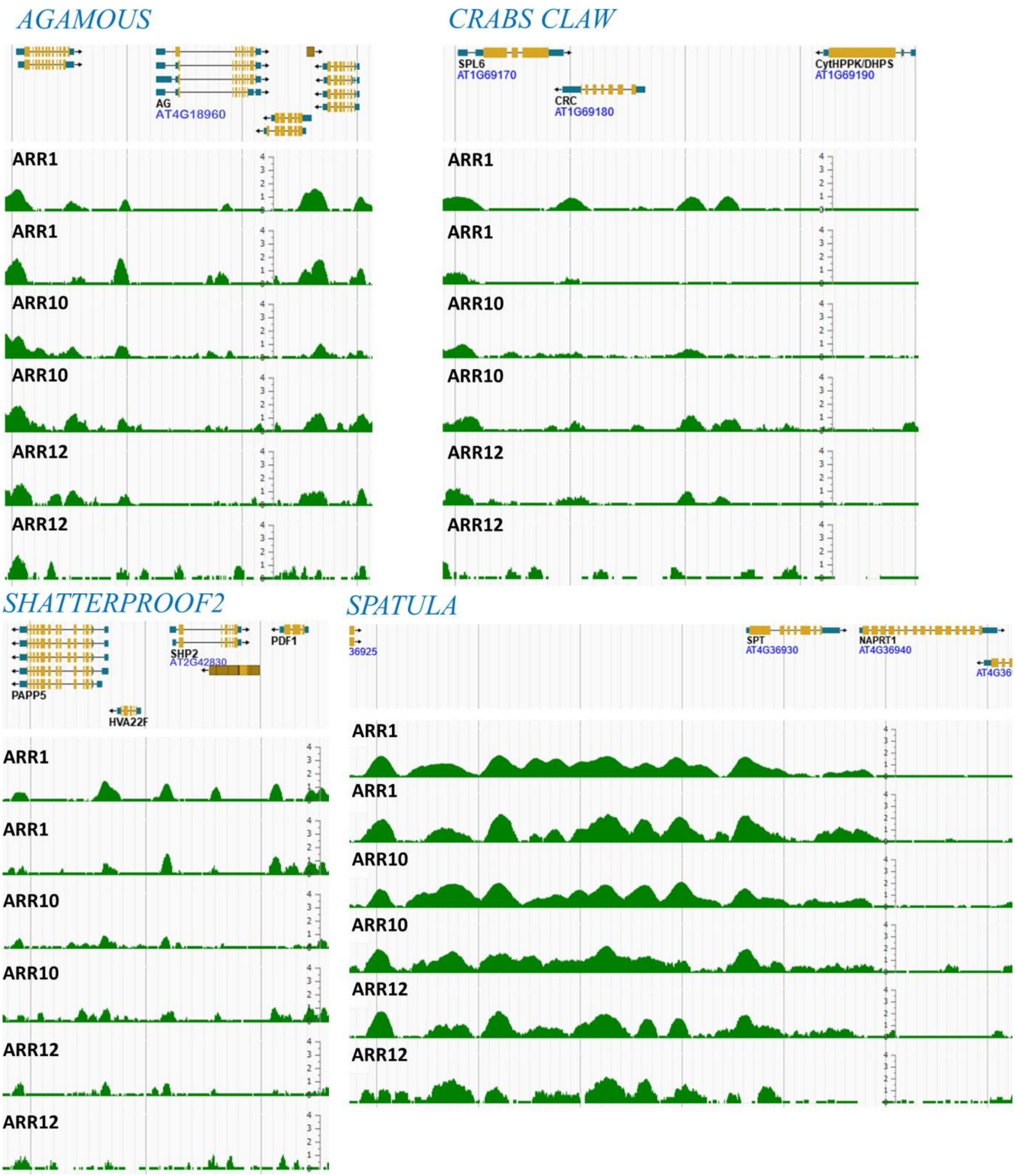


**Figure S1**. Type-B ARR binding events in the *AG*, *CRC*, *SPT* and *SHP2* regulatory regions. Visualization of ChIP-seq tracks of ARR1, ARR10, and ARR12 (replicates; 3-day seedlings) reported by Xie *et al*., (2018), using the Encyclopedia of Plant Genome (ENPG; www.plantseq.org). The intensity of ChIP-seq signals is presented as the height of peaks on a log scale.

**Table S1.** Oligonucleotides used in this study.

| Gene alias | Gene ID | Experiment | Primer name | Sequence | Fragment Length (bp) |
|---|---|---|---|---|---|
| AG | AT4G18960 | qRT-PCR | SDF1159 | ACCAGATTCTTCGTGCAAAGATAGCTG | 27 |
| | | | SDF1160 | AAGCTGCTCGTAGTTAGATCCTCCTG | 26 |
| ARR7 | AT1G19050 | qRT-PCR | SDF1155 | TGCCAGGACTTTCAGGATATGATCTCC | 27 |
| | | | SDF1156 | ACAAGAATTCCTCTGCTCCTTCTTTGAG | 28 |
| SHP2 | AT2G42830 | qRT-PCR | SDF973 | TGTCTTGTGTGACGCTGAGGTT | 22 |
| | | | SDF974 | GTACCTTTCTATTGTTCCTCTCACACTGT | 29 |
| CRC | AT1G69180 | qRT-PCR | SDF969 | CCCTCAAGCCGAACATCTCTA | 21 |
| | | | SDF970 | CACCGTTACCGTGTCAAGCA | 20 |
| SPT | AT4G36930 | qRT-PCR | SDF975 | TGTGGCTTCTTCATACGCTTTG | 22 |
| | | | SDF976 | TAACCCACCTTCCCGACTCA | 20 |
| ACT2 | AT3G18780 | qRT-PCR | SDF1161 | AATCACAGCACTTGCACC | 18 |
| | | | SDF1162 | ATTCCTGGACCTGCCTC | 17 |
